# Supplementary material for: Negative Dielectric Anisotropy Liquid Crystal with Improved Photo-Stability, Anti-Flicker, and Transmittance for 8K Display Applications
Source: Molecules. 2022 Oct 22;27(21):7150. doi: 10.3390/molecules27217150 (PMC9659119; doi:10.3390/molecules27217150)
Supplement: Supplementary file 1 [file molecules-27-07150-s001.zip › molecules-1955117-supplementary.pdf]

Support information

Negative dielectric anisotropy liquid crystal with improved photo-stability, anti-Flicker, and transmittance for 8K display applications

Haiguang Chen[1,2,3], Youran Liu[2,3], Maoxian Chen[2,3], Tianmeng Jiang[2,3], Zhou Yang[1,*], Huai Yang[4,5,*]

[1] School of Materials Science and Engineering, University of Science and Technology Beijing, China.

[2] Beijing Bayi Space Liquid Crystal Material Technology Company.

[3] Beijing Key Laboratory of Liquid Crystal Materials Analysis and Application Technology, Beijing, China.

[4] Department of Materials Science and Engineering, College of Engineering, Peking University, Beijing, China.

[5] Key Laboratory of Polymer Chemistry and Physics of Ministry of Education, Peking University, Beijing, China

* Correspondence: yangz@ustb.edu.cn (Z.Y); yanghuai@pku.edu.cn (H.Y); Tel.: 01062333759 (Z.Y); 010-62766919 (H.Y)

Table S1. Composition and content of liquid crystal A

Enty	Structural	Content (%)
1	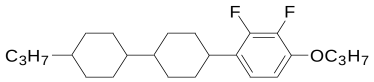	7
2	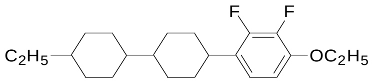	6
3	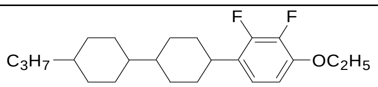	8
4	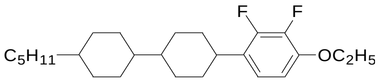	8
5	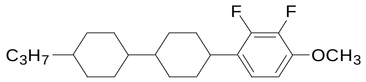	8
6	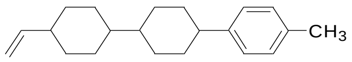	7
7	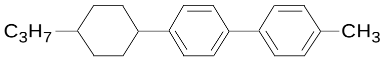	6
8	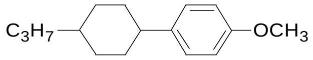	9
9	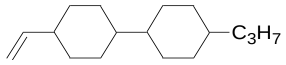	25
10	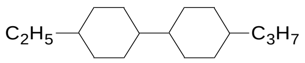	16

Table S2. The structure of Type B negative dielectric anisotropy compounds.

Enty	Structural
b1	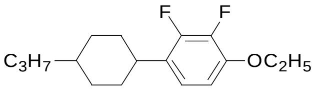
b2	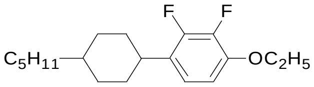
b3	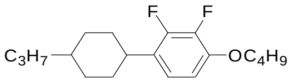

Table S3. The structure of Type C negative dielectric anisotropy compounds.

Enty	Structural
c1	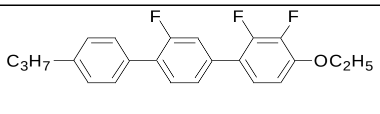
c2	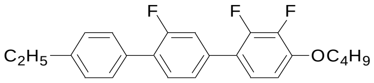
c3	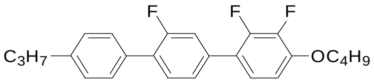

Table S4. The structure of Type D negative dielectric anisotropy compounds.

Enty	Structural
d1	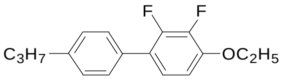
d2	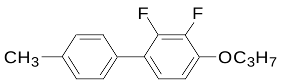
d3	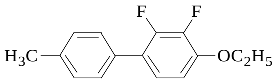
d4	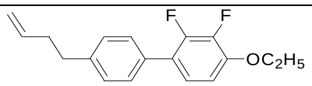

Table S5. The structure of Type E negative dielectric anisotropy compounds.

Enty	Structural
e1	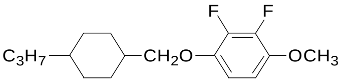
e2	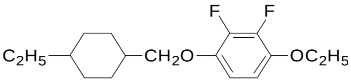
e3	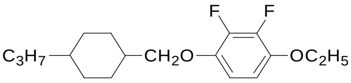
e4	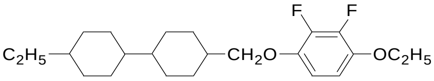
e5	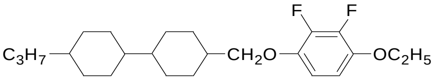
e6	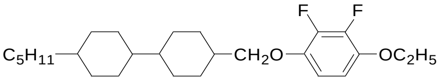
e7	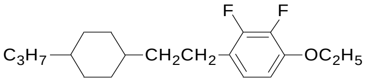

Table S6. The structure of Type F negative dielectric anisotropy compounds.

Enty	Structural
f1	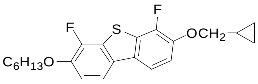
f2	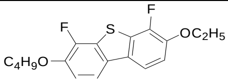
f3	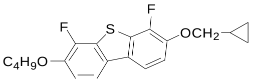
f4	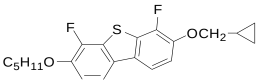
f5	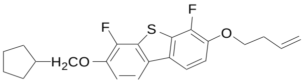

f6	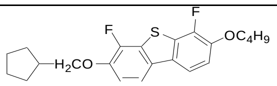

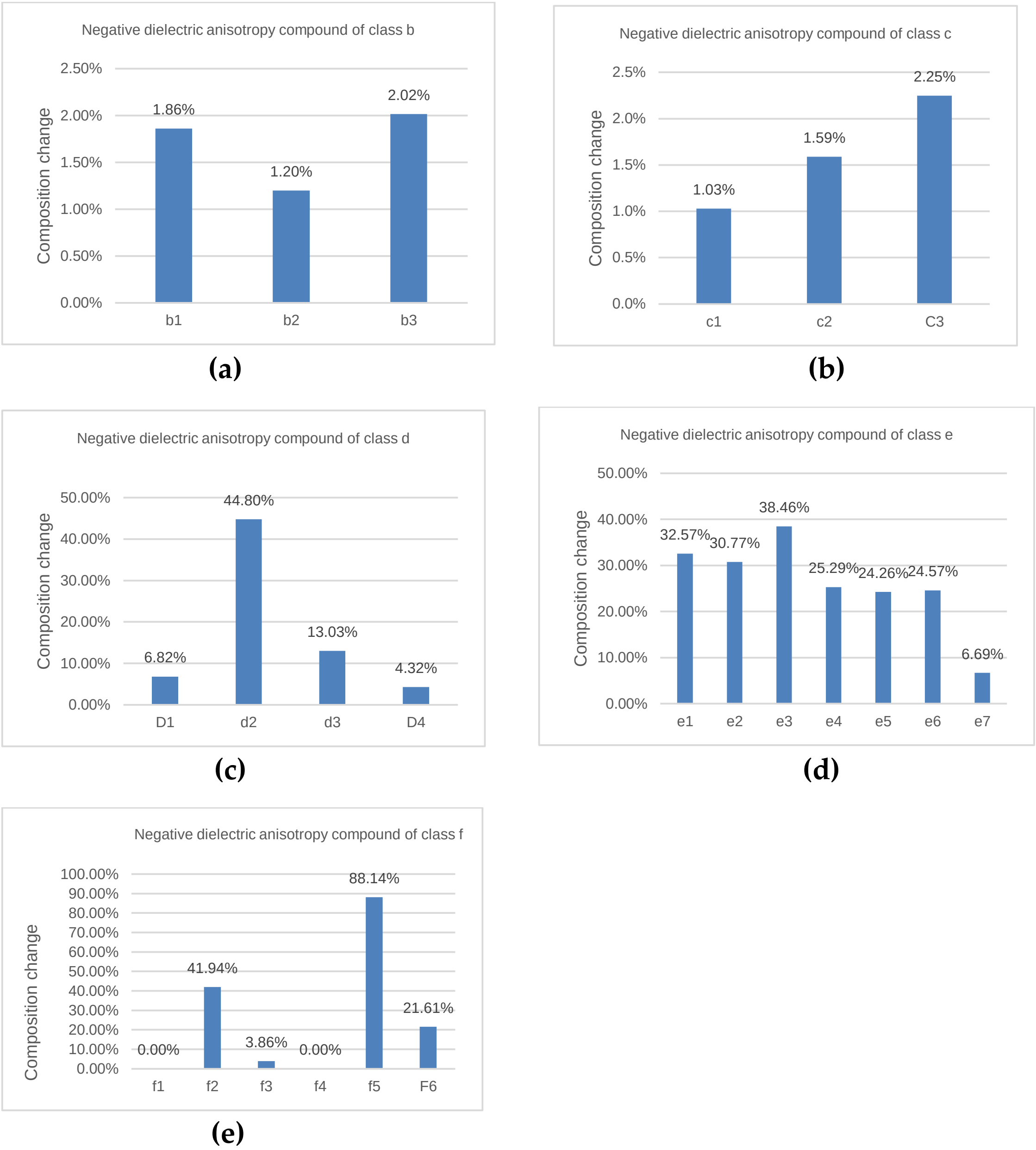

Figure S1. (**a**) The composition change of Type B negative dielectric anisotropy compounds. (**b**) The composition change of Type C negative dielectric anisotropy compounds. (**c**) The composition change of Type D negative dielectric anisotropy compounds. (**d**) The composition change of Type E

negative dielectric anisotropy compounds. (**e**) The composition change of Type F negative dielectric anisotropy compounds.

Table S7. Content of phenolic components under two conditions

	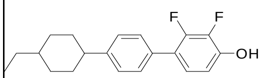	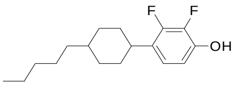	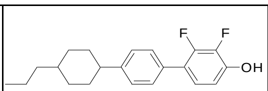	39.53
mixed liquid crystal B	0.045	0.043	0.034	1.000
mixed liquid crystal B + anti-light stabilizer	0.028	0.029	0.042	1.000

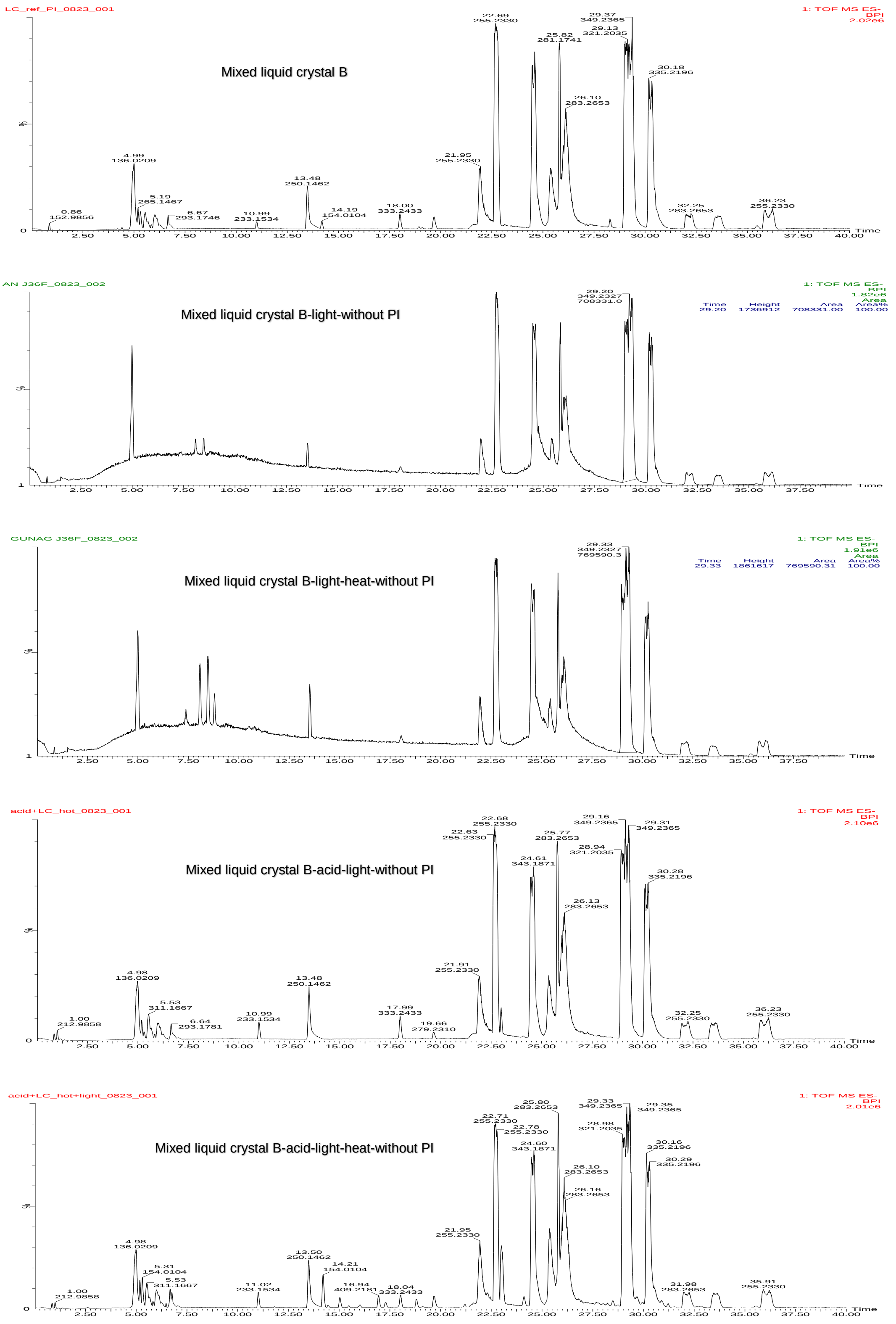

Figure S2. Content of phenols in different test environments without PI.

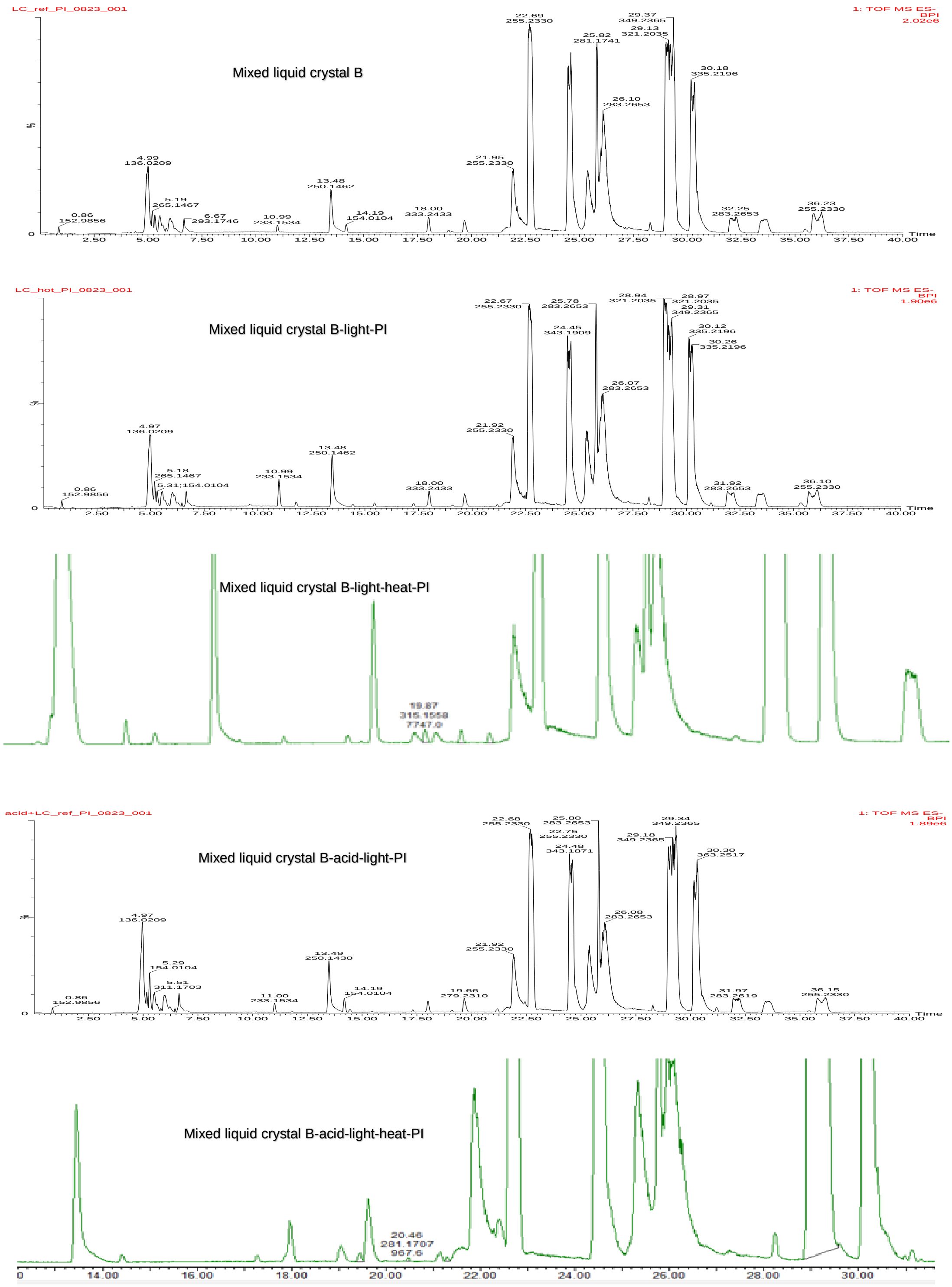

Figure S3. Content of phenols in different test environments with PI.

Table S8. Composition and content of liquid crystal C

Enty	Structural	Content (%)
1	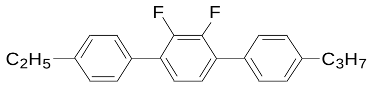	1.5
2	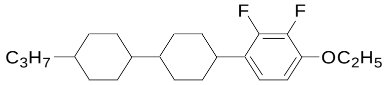	6
3	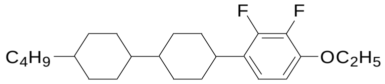	4
4	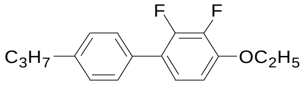	10
5	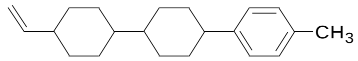	5
6	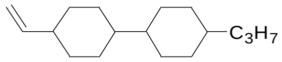	40
7	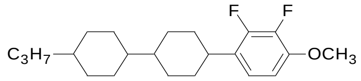	5
8	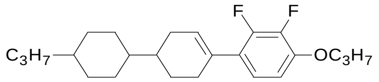	5
9	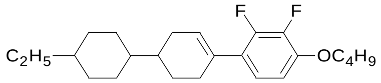	5.5
10	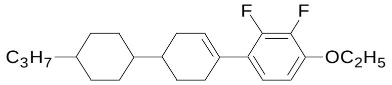	7
11	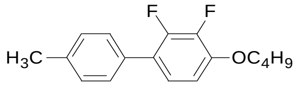	3
12	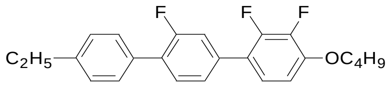	2
13	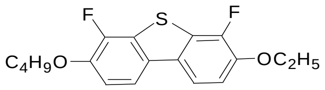	3
14	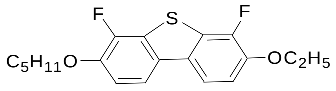	3

Table S9. Physical properties of mixed liquid crystal C.

**Liquid Crystal Property**		
Clear point	T_{NI} [°C]	81.0
Dielectric anisotropy [25°C, 1kHz]	$\triangle\varepsilon$	-4.05
	$\varepsilon_{\parallel}$	3.85
	$\varepsilon_{\perp}$	7.90
Elastic constant [25°C]	K_{11}[pN]	14.60
	K_{22}[pN]	7.30
	K_{33}[pN]	14.40
Rotary Viscosity[25°C]	γ1[mPa.s]	80.0
Optical anisotropy [589nm,25°C]	n_e	1.5943
	n_o	1.4856
	$\triangle$n	0.1087

Table S10. Ion content of mixed liquid crystal C and D.

Item	VHR/%	ION/pC
Mixed liquid crystal C	83.5	436.5
Mixed liquid crystal D	88.4	302.6

Ta**ble S11**. Ion content of mixed liquid crystal D and E.

Item	VHR/%	ION/pC
Mixed liquid crystal D	88.4	302.6
Mixed liquid crystal E	88.2	302.8

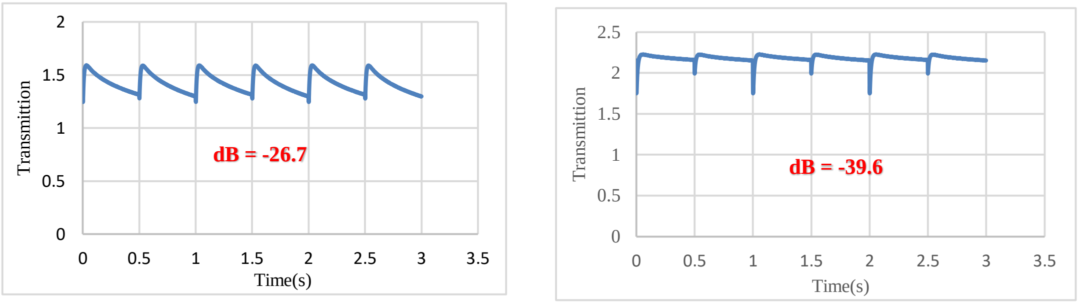

(a) (b)

Figure S4. (a) Transmission and Flicker of liquid crystal D. (b) Transmission and Flicker of liquid crystal E

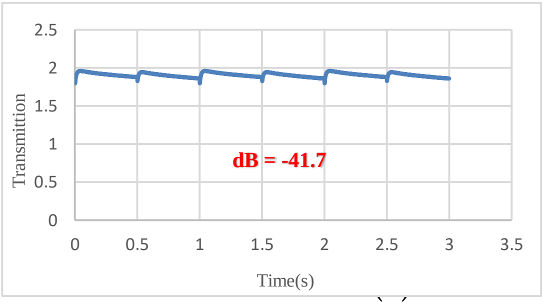

Figure S5. Transmission and Flicker of liquid crystal F

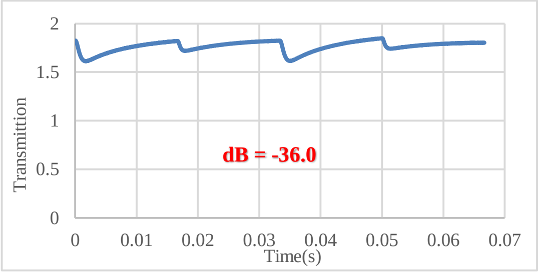

(a)

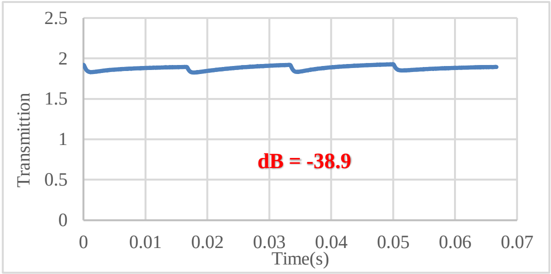

(b)

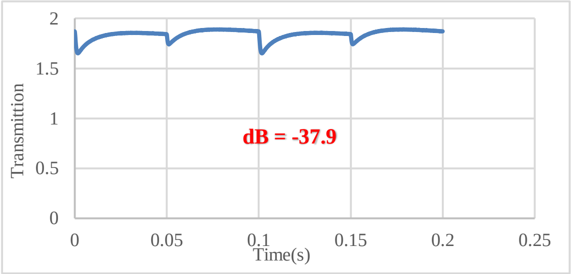

(c)

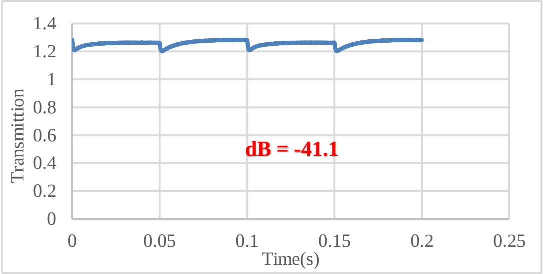

(d)

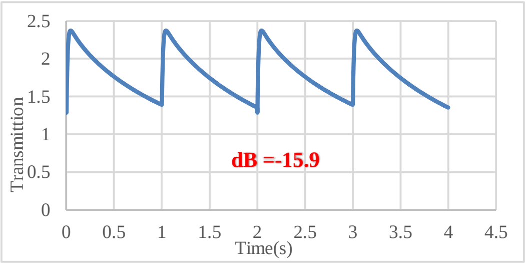

(e)

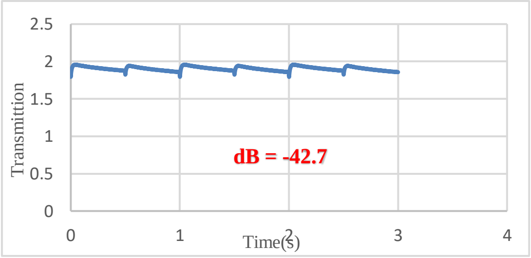

(f)

Figure S6. (a) Transmission and Flicker of mixed liquid crystal C at 30Hz . (b) Transmission and Flicker of mixed liquid crystal G at 30Hz. (c) Transmission and Flicker of mixed liquid crystal C at 10Hz . (d) Transmission and Flicker of mixed liquid crystal G at 10Hz. (e) Transmission and Flicker of mixed liquid crystal C at 0.5Hz . (f) Transmission and Flicker of mixed liquid crystal G at 0.5Hz.

Table S12. Relationship between Structure and performance of Liquid Crystal

parameters	Adjustment	Monomer structure
$\gamma 1$	↑	Cyclohexane，Long carbon chain
	↓	Phenyl，Dioxane，Alkenyl，Linking group
K11	↑	Cyclohexane，Polycyclic
	↓	Phenyl
K22	↑	Cyclohexane，Polycyclic
	↓	Phenyl
K33	↑	Cyclohexane，Polycyclic，Alkenyl
	↓	Phenyl

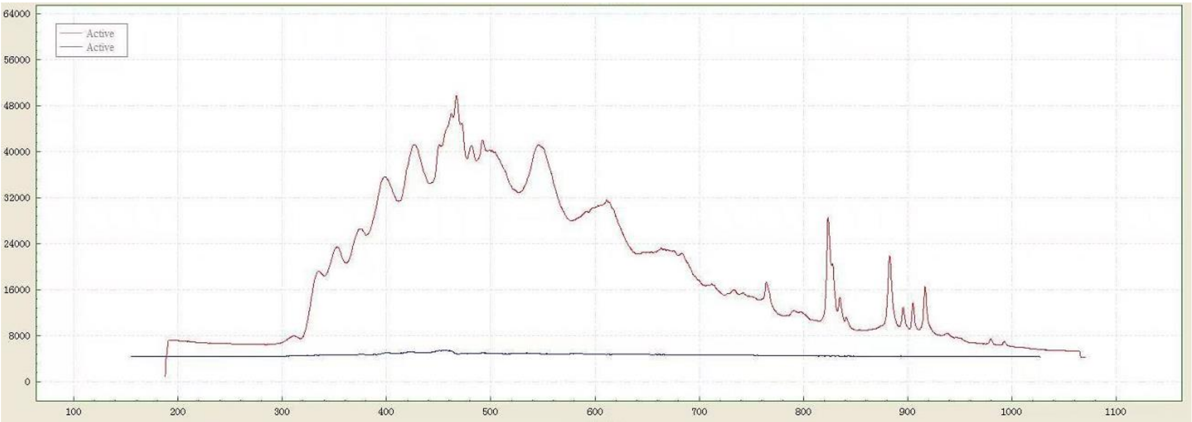

Figure S7. The spectral of the backlight.
